# Supplementary material for: Causal Relationship between Aging and Anorexia Nervosa: A White-Matter-Microstructure-Mediated Mendelian Randomization Analysis
Source: Biomedicines. 2024 Aug 16;12(8):1874. doi: 10.3390/biomedicines12081874 (PMC11351342; doi:10.3390/biomedicines12081874)
Supplement: Supplementary file 1 [file biomedicines-12-01874-s001.zip › Supplementary Figure/Figure S9/Leaveoneout plot of MR analysis for ubm-b-1874 to AN.pdf]

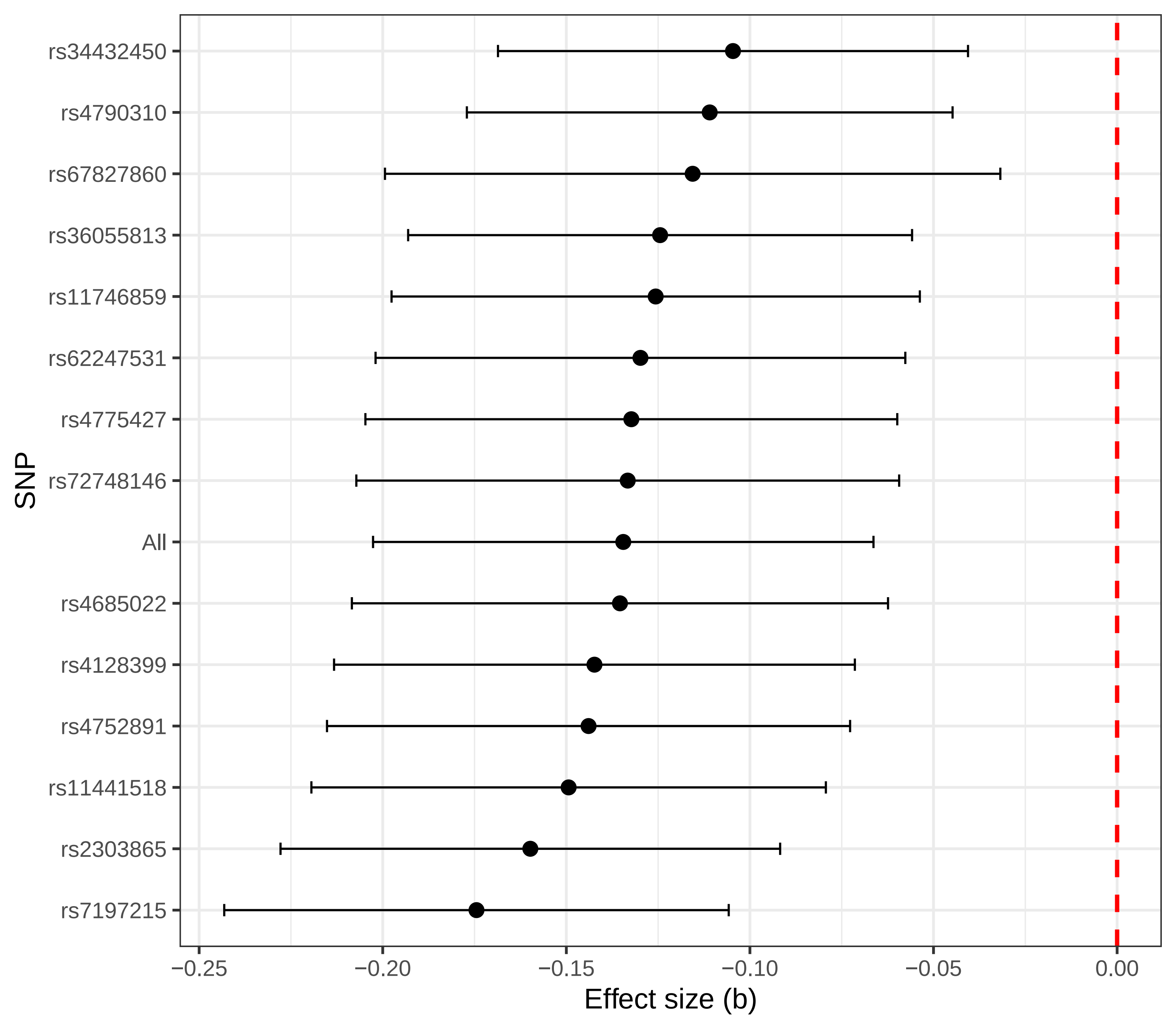

rs34432450
rs4790310
rs67827860
rs36055813
rs11746859
rs62247531
rs4775427
rs72748146
All
rs4685022
rs4128399
rs4752891
rs11441518
rs2303865
rs7197215
SNP
−0.25
−0.20
−0.15
−0.10
−0.05
0.00
Effect size (b)
